# Prioritizing determinants of cognitive function in healthy middle-aged and older adults: insights from a machine learning regression approach in the Canadian longitudinal study on aging

**DOI:** 10.3389/fpubh.2023.1290064

**Published:** 2023-12-22

**Authors:** Sarah Singh, Shiran Zhong, Kem Rogers, Vladimir Hachinski, Stephanie Frisbee

**Affiliations:** ^1^Robarts Research Institute, University of Western Ontario, London, ON, Canada; ^2^Department of Geography, University of Western Ontario, London, ON, Canada; ^3^Department of Anatomy and Cell Biology, Schulich School of Medicine and Dentistry, University of Western Ontario, London, ON, Canada; ^4^Department of Clinical Neurological Sciences, and Epidemiology and Biostatistics, Schulich School of Medicine and Dentistry, University of Western Ontario, London, ON, Canada; ^5^Department of Pathology and Laboratory Medicine, and Epidemiology and Biostatistics, Schulich School of Medicine and Dentistry, University of Western Ontario, London, ON, Canada

**Keywords:** cognitive function, determinants of health, dementia prevention, machine learning, CLSA

## Abstract

**Introduction:**

The preservation of healthy cognitive function is a crucial step toward reducing the growing burden of cognitive decline and impairment. Our study aims to identify the characteristics of an individual that play the greatest roles in determining healthy cognitive function in mid to late life.

**Methods:**

Data on the characteristics of an individual that influence their health, also known as determinants of health, were extracted from the baseline cohort of the Canadian Longitudinal Study of Aging (2015). Cognitive function was a normalized latent construct score summarizing eight cognitive tests administered as a neuropsychological battery by CLSA staff. A higher cognitive function score indicated better functioning. A penalized regression model was used to select and order determinants based on their strength of association with cognitive function. Forty determinants (40) were entered into the model including demographic and socioeconomic factors, lifestyle and health behaviors, clinical measures, chronic diseases, mental health status, social support and the living environment.

**Results:**

The study sample consisted mainly of White, married, men and women aged 45–64 years residing in urban Canada. Mean overall cognitive function score for the study sample was 99.5, with scores ranging from 36.6 to 169.2 (lowest to highest cognitive function). Thirty-five (35) determinants were retained in the final model as significantly associated with healthy cognitive functioning. The determinants demonstrating the strongest associations with healthy cognitive function, were race, immigrant status, nutritional risk, community belongingness, and satisfaction with life. The determinants demonstrating the weakest associations with healthy cognitive function, were physical activity, greenness and neighborhood deprivation.

**Conclusion:**

Greater prioritization and integration of demographic and socioeconomic factors and lifestyle and health behaviors, such greater access to healthy foods and enhancing aid programs for low-income and immigrant families, into future health interventions and policies can produce the greatest gains in preserving healthy cognitive function in mid to late life.

## 1 Introduction

Optimal cognitive functioning, broadly defined as the adequate processing and application of knowledge, is essential to healthy living and successful aging (1, 2). Research has demonstrated that the risk of poor cognitive functioning, otherwise known as cognitive impairment, increases exponentially with age (3). Given the aging population in Canada, 956,000 seniors are projected to be living with dementia, a severe form of cognitive impairment, by the year 2030 (4). Dementia is a debilitating and costly condition that involves an array of medical services, including but not limited to hospitalization, nursing care, in-home assistance, physical therapy and prescription drugs. Consequently, dementia has cost the Canadian economy approximately $12 billion in 2021 (5). Therefore, the prevention of dementia, through the early preservation of cognitive function, has become a top public health priority (6).

Healthy cognitive function is determined by multiple factors, including our personal characteristics and the environments in which we live and work, also known as determinants of health. In 2015, researchers at the University of Wisconsin Population Health Institute, in collaboration with the Robert Wood Johnson Foundation, sought to rank the health of geographic counties in the US and examine the contribution of modifiable determinants of health to these rankings (7). The study produced the well-known County Health Rankings Model which indicated that the health of counties, measured by quality and length of life, were determined according to the following contributions: 40% from social and economic factors, 30% from health behaviors, 20% from clinical care, and 10% from the physical environment. Authors concluded that determinants exerting the most powerful influence on health outcomes were social and economic factors (8). Almost a decade later, despite these key findings, much of the healthcare spending remains allocated to clinical care and pharmaceutical services.

From a population health perspective, there exists major challenges in designing preventive interventions aimed at preserving cognitive health. Given recent developments in data analytics and big data, new determinants of health continue to emerge rapidly (9). This growth has outpaced our ability to successfully process and implement strategies that effectively incorporate novel determinants into current health interventions (10). Furthermore, there remains a lack of rigorous scientific evidence to prioritize determinants for knowledge translation and implementation purposes (11). The prioritization of determinants identifies those areas that are highly amenable to intervention, that is, feasible, cost effective and substantially reduces disease burden in the population (12). While prioritization may seem a sizeable task, an important next step in research is to quantify, sort and compare the effects of a range of modifiable and non-modifiable determinants on health. Such findings would guide knowledgeable investment into health programs and policy change that target specific key determinants of health.

As the County Health Ranking Model posits, various determinants contribute to the health of individuals (7). Often researchers have studied these determinants in isolation rather than collectively. A reason for this phenomenon may be the limitation of including correlated factors, measuring similar dimensions, in the same traditional statistical model. Accordingly, advances in machine learning algorithms have provided more opportunities to examine multiple determinants of health in the same regression model (13). Specifically, machine learning regression approaches can successfully reduce a model with many determinants to a smaller set of only the strongest determinants. Published research has indicated that, when applied correctly, machine learning regression methods perform with high accuracy and provide robust estimates in comparison to traditional statistical models (14–16).

Given the multifactorial nature of cognitive function, the process of identifying specific determinants with the greatest impact would be key to informing future interventions seeking to preserve cognitive functioning in healthy individuals. Such a process does not aim to rule out causes of cognitive impairment in individuals, but instead, highlights target areas that can improve or preserve cognitive health in the entire population. Therefore, the aim of this study was to employ a machine learning penalized regression method to identify and select determinants of health that play the greatest roles in determining cognitive function in healthy adults.

## 2 Methods

Using baseline data from the Canadian Longitudinal Study of Aging, our study employs a machine learning penalized regression approach to identify and select determinants of health, according to their strength of association with healthy cognitive functioning, in a sample of middle-aged and older adults without known cognitive impairment.

### 2.1 Study sample and data source

The Canadian Longitudinal Study Aging (CLSA) is a longitudinal follow-up study on ~50,000 adults aged 45–85 years across Canada. Data was collected on demographic and health-related data of healthy individuals nationally. Baseline data collection was completed in 2015 and participants continue to be followed at 3-year intervals within a 20-year study period. Our study focused on the baseline data from the Comprehensive cohort of the CLSA, which consisted of 30,097 individuals who underwent in-person interviewing, site visit testing and cognitive testing. Participants were recruited from provincial health registries and random digit telephone dialing. The CLSA excluded participants as follows: inability to communicate in either English or French, not residing in one of the 10 provinces, residing on First Nation reserve or settlement, institutionalized, serving member of the Canadian Armed Forces, exhibited cognitive impairment at the time of recruitment.

Cognitive testing in CLSA consisted of standardized, evidence-based, and clinically relevant indicators of cognitive performance for consenting participants of the CLSA. Exclusions from our study included any participant missing data for the study outcome on overall cognitive function and for testing elements used to create the outcome variable. Thus, the final sample size for our study was 25,168. A full description of the CLSA, including study design, recruitment and instruments is publicly available for review (17). Given our use of anonymized secondary data for this study, this study qualifies for exempt from the ethics board at Western University. The application for the use of CLSA data was approved by the CLSA on January 29, 2022 (Application #2109031).

### 2.2 Study outcomes

The outcome for this study was cognitive function measured by a latent construct score for overall cognition developed by the CLSA (18). The overall cognition latent construct score is based on scores achieved in eight cognitive tests administered as a neuropsychological battery to study participants by CLSA staff. For the comprehensive cohort, the following eight cognitive tests were performed both in person and via interviewing: Rey Auditory Verbal Learning Test immediate recall (REY I) and five-min delayed recall (REY II), Mental Alternation Test (MAT) for speeded alternation of ascending letters and numbers, Animal Fluency (AFT) for generative verbal fluency, FAS for generative phonemic fluency for the letters F, A, and S, Victoria Stroop Test (STP), a time-based prospective memory task (TMT total score) and event-based prospective memory task (PMT total score) (19). Testing was focused on memory and executive function.

The main outcome for this study was an overall cognition latent construct score developed by the CLSA and made available in the comprehensive assessment of the baseline study cohort. Briefly, for each neuropsychological test, a normed score was created using regression based models with stratification by age, sex and education. Normed scores were combined with multi-group confirmatory factor analysis to create an overall cognition latent score scaled to a mean of 100 with a standard deviation on 15. A higher latent score indicated better cognitive functioning. The methodology used to create the overall cognition latent score, including the justification for each cognitive test used in the CLSA, along with descriptive statistics for the distribution of cognitive test scores in the CLSA, have been published elsewhere (18, 20, 21).

### 2.3 Study determinants

Data on determinants were attained by the CLSA through in-person interviews for the comprehensive cohort, except in rare cases where participants could not be interviewed in-person. A total of 40 determinants of health were extracted from the CLSA database for the purposes of this study. The selection of determinants was guided by the four categories of determinants proposed in the County Health Ranking Model and adapted for assessing associations with cognitive function. For the purposes of this study, determinants were theoretically grouped into seven (7) categories: Demographics and Socioeconomic Determinants, Clinical Determinants, Chronic Disease Determinants, Lifestyle and Behavioral Determinants, Mental Health Determinants, Social Support Determinants, and Living Environment Determinants. A full outline of categories and determinants is shown in Table 1.

**Table 1:** List of 40 study determinants of health extracted from baseline cohort of the Canadian Longitudinal Study of Aging, Baseline Cohort 2015 (CLSA).

**Categories of study determinants**						
**Demographics and socioeconomic**	**Clinical**	**Chronic disease**	**Lifestyle and behavioral**	**Mental health**	**Social support**	**Living environment**
**List of Study determinants**						
• Age • Race • Marital status • Sex • Urban/rural area of residence • Education • Income • Immigration status • Access to primary care	• Blood systolic pressure • Blood diastolic pressure • Total cholesterol • Non-HDL • HBA1c	• Heart disease • Peripheral vascular disease • Cancer • Kidney disease • Diabetes mellitus • Hypertension • Angina • Acute myocardial infarction • Stroke	• Smoking • Alcohol consumption • Nutrition status • Body mass index • Physical activity • Sleep duration	• Depression • Satisfaction with Life	• Affectional Support • Emotional Support • Social Support • Tangible Support • Sense of community belonging	• Greenness • Material deprivation • Social deprivation • Active living index

#### 2.3.1 Demographics and socioeconomic determinants

Variables within this category described the self-reported demographic and socioeconomic characteristics of an individual and include age, race, marital status, sex, urban/rural area of residence, education and income, immigration status. Age was represented as age in years at baseline categorized as 45–54, 55–64, 65–74 and 75 years and over. Race was represented as cultural background categorized as “White” or “Non-White”. Marital status was categorized as single, married/common-law, widowed, divorced or separated. Sex was categorized as male or female. Area of residence was categorized as rural, urban core, urban fringe, urban population outside census metropolitan areas and agglomerations or secondary core. Education was categorized as less than secondary school, secondary school, some post secondary, post secondary degree/diploma education. Income was represented as total household income from all sources before taxes and categorized as <$20,000, $20,000–49,999, $50,000–99,999, $100,000–149,999, $150,000 or more. Immigration status was categorized as whether the participant identified with being an immigrant or non immigrant to Canada. Access to care was assessed as whether or not the respondent reported having a primary care physician.

#### 2.3.2 Clinical determinants

Variables within this category described measurements conducted at data collection sites for the CLSA and include blood pressure, blood cholesterol, and blood glucose. All measurements were taken using standard operating clinical procedures (17). Blood pressure was represented as the average systolic and diastolic blood pressures (mmHg) taken over six readings, excluding the first reading. Blood cholesterol was represented as total blood cholesterol (mmol/L) and blood non-High-Density Lipoprotein (mmol/L). Blood glucose was represented as non-fasting HBA1c (%).

#### 2.3.3 Chronic disease determinants

Variables within this category described the self-reported presence of chronic diseases. During interviews, participants were asked whether they had been told by a doctor that they had any of the following conditions: heart disease, peripheral vascular disease, cancer, kidney disease, diabetes mellitus, hypertension, angina, stroke or acute myocardial infarction.

#### 2.3.4 Lifestyle and behavioral determinants

Variables within this category describe self-reported and measured health behavior and lifestyle choices. During interviews, participants were asked about smoking, alcohol consumption, nutrition status, body mass index, physical activity, sleep duration, and access to care. Smoking was represented whether participants smoked more than 100 cigarettes in their lifetime. Alcohol consumption was represented as frequency of alcohol consumption; almost daily, 4–5 times weekly, 2–3 times weekly, weekly, 2–3 times a month, once a month, less than once a month, never.

Nutrition status was categorized as high or low nutritional risk. Nutritional risk is measured in the CLSA using AB SCREENTM II (Abbreviated Seniors in the Community Risk Evaluation for Eating and Nutrition II) (22). The tool uses eight self reported questions on weight change and meal preparation. The nutritional risk score ranges from 0 to 48, with lower scores indicating higher risk. A nutritional risk score of <38 indicated high nutritional risk. According to the CLSA protocol, the AB SCREEN™ II assessment tool is owned by Dr. Heather Keller and the use of the AB SCREEN™ II assessment tool was made under license from the University of Guelph for the purposes of the study.

Body mass index was calculated by CLSA using measured data on height and weight collected as data collection sites. Physical activity was determined by CLSA in interviews using the previously validated Physical Activity Scale for the Elderly (PASE), designed to assess the duration, frequency, exertion level, and amount of physical activity over a seven-day period by individuals 65 years and older (23). PASE score ranging from 0 to 793, with higher scores indicating greater physical activity. Physical activity was represented by the respondent PASE score. Sleep was represented as the reported number of hours of sleep on average per night in the past month.

#### 2.3.5 Mental health determinants

Variables within this category describe mental health status. During interviews, participants were assessed for depression and satisfaction with life. Depression was assessed using the Center for Epidemiological Studies Short Depression Scale (CES-D). Depression was represented as the respondent's CES-D 10 score. The CES-D 10 is a 10-item Likert scale questionnaire assessing depressive symptoms in the past week and the final score is a sum of the 10-item responses. The final CES-D 10 score ranged from 0 to 30 with higher scores suggesting greater severity of symptoms (24). Based on the score, participants were categorized as depressed or not depressed using a cutoff point of 10. Satisfaction with life was assessed using the Satisfaction With Life Scale (SWLS) (25). Satisfaction with life was represented as the respondent's SWLS Score which is an aggregate score of the responses to the five items of the SWLS. Individual responses to each item in the SWLS range from 1—strongly disagree to 7—strongly agree. Higher scores indicate a greater satisfaction with life. Participants were placed into the following categories based in their SWLS Score: extremely dissatisfied (5-9), dissatisfied (10-14), slightly dissatisfied (15-19), neutral (20), slightly satisfied (21-25), satisfied (26-30), extremely satisfied (31-35) (26).

#### 2.3.6 Social support determinants

Variables within this category describe participants' perception of received social support and community belongingness. Social support was assessed using the 19-item Medical Outcomes Study (MOS) Social Support Survey and represented as MOS scores for the following four subscales: Affectional, Emotional and Informational, Positive Social and Tangible (27). A transformed score was obtained for each subscale from CLSA and used as independent determinants in analyses. Community belongingness was assessed as participants' agreement or disagreement with whether they felt a sense of belonging to their community of residence.

#### 2.3.7 Living environment determinants

Variables within this category describe the living environment or neighborhood in which respondents reside. The CLSA employed validated measures of the living environment through linkage with (The Canadian Urban Environmental Health Research Consortium) data. Living environment was assessed using the average annual normalized difference vegetation index (NDVI), neighborhood deprivation and active living. Estimates of greenness were based on the remotely sensed NDVI, assigned by CLSA, using the centroid location of each participant's six-character residential postal code (28). The NDVI values range from −1 to 1, with negative values representing water, values around zero (−0.1 to 0.1) representing bare soil or impervious surfaces, and higher positive values representing dense green vegetation. The NDVI metrics, indexed to DMTI Spatial Inc. postal codes, were provided by CANUE (The Canadian Urban Environmental Health Research Consortium) (29–33). NDVI data from 2011–2013 was provided by CLSA and used in this study.

Neighborhood deprivation was assessed using indices on material and social deprivation. Material deprivation was assessed based on the proportion of individuals without a high school diploma, the employment-to-population ratio, and the average personal income of individuals. Social deprivation was assessed based on the proportion of people who live alone, are separated, divorced or widowed, or are a lone parent. Data on material and social deprivation were available as quintiles with the highest quintile representing the most deprivation. Material and Social Deprivation Indices (MSDI), indexed to DMTI Spatial Inc. postal codes, were provided by CANUE (Canadian Urban Environmental Health Research Consortium) Material and Social Deprivation Indices (MSDI) used by CANUE were provided by: Institut National de Santé Publique du Québec (INSPQ). Indices were compiled for 1991, 1996, 2001, and 2011 Census data by the Bureau d'information et d'études en santé des populations (BIESP) (29, 34).

Active living was measured by the Canadian Active Living Environments Database (ALE) to indicate the walkability of neighborhoods. For the purposes of this study, we utilized the active living environment class which is a categorical value characterizing the favourability of the ALE on a scale from 1 (very low) to 5 (very high) (29, 35). Canadian Active Living Environments Index (Can-ALE), indexed to DMTI Spatial Inc. postal codes, were provided by CANUE (Canadian Urban Environmental Health Research Consortium).

### 2.4 Missing data

Individuals from the CLSA sample who were missing data on the study outcome or testing that comprised the outcome were excluded from this study. The CLSA reported missing values on cognitive tests if the participant was unable to complete the required tasks of the tests or did not consent to the testing, or if the results of the test were not interpretable. Missing data on determinants occurred if the participant refused to answer the interview question or if the blood sample testing was not completed accurately. Individuals who refused to answer interview questions on determinants or individuals without completed blood sample testing were not excluded from the study. Their responses were categorized as missing and retained in analyses as shown in Table 2. Missing data on determinants were low (10% or less) thus imputation was not employed.

**Table 2:** Characteristics of the study sample based on study determinants of health, Canadian Longitudinal Study of Aging Baseline Cohort 2015.

**Study determinants**		**Frequency (%) mean (s.d.); *N* = 25,168**	**Correlation with study outcome**
**Demographic and socioeconomic determinants**			
Age (years)	45–54	6,584 (26.2)	**< 0.0001**
	55–64	8,315 (33.0)	
	65–74	6,046 (24.0)	
	75 and older	4,223 (16.8)	
Race	Non-White	1,093 (4.3)	**< 0.0001**
	White	24,075 (95.7)	
Marital status	Single	2,167 (8.6)	**< 0.0001**
	Married/common-law	17,442 (69.3)	
	Widowed	2,270 (9.0)	
	Divorced	2,623 (10.4)	
	Separated	660 (2.6)	
	Missing	6 (0.02)	
Sex	Female	12,922 (51.3)	0.98
	Male	12,246 (48.7)	
Urban/rural area of residence^*^	Rural	1,996 (7.9)	**0.0009**
	Urban core	21,829 (86.7)	
	Urban fringe	471 (1.9)	
	Urban population	170 (0.7)	
	Urban secondary core	390 (1.6)	
	Missing	312 (1.2)	
Education	Less than secondary	1,312 (5.2)	0.29
	Some post secondary	2,331 (9.4)	
	Graduated secondary	1,873 (7.3)	
	Post secondary	19,631 (78.1)	
	Missing	21 (0.08)	
Income	< $20,000	1,251 (5.0)	**< 0.0001**
	$20,000–49,999	5,226 (20.8)	
	$50,000–99,999	8,332 (33.1)	
	$100,000–149,999	4,744 (18.8)	
	$150,000 or more	4,148 (16.5)	
	Missing	1,467 (5.8)	
Immigrant status	Yes	4,468 (17.8)	**< 0.0001**
	No	20,677 (82.2)	
	Missing	23 (0.09)	
Access to primary care	Yes	21,743 (86.4)	**0.01**
	No	2,350 (9.3)	
	Missing	1,075 (4.3)	
**Clinical determinants**			
Blood systolic pressure (mmHg)		122.1 (16.9)	**< 0.0001**
	Missing	121 (0.5)	
Blood diastolic pressure (mmHg)		74.4 (9.9)	**0.02**
	Missing	121 (0.5)	
Total cholesterol (mmol/L)		5.2 (1.1)	**< 0.0001**
	Missing	2,483 (10.0)	
Non-HDL (mmol/L)		3.7 (1.0)	**0.0008**
	Missing	2,483 (10.0)	
HbA1c (%)		5.2 (1.0)	**< 0.0001**
	Missing	2,554 (10.1)	
**Chronic disease determinants**			
Heart disease	Yes	2,821 (11.3)	**0.003**
	No	22,218 (88.7)	
	Missing	129 (0.5)	
Peripheral artery disease	Yes	1,316 (5.2)	**0.02**
	No	23,733 (94.8)	
	Missing	119 (0. 5)	
Cancer	Yes	3,858 (15.4)	0.08
	No	21,256 (84.6)	
	Missing	54 (0.2)	
Kidney disease	Yes	687 (2.7)	0.08
	No	24,392 (97.3)	
	Missing	89 (0.4)	
Diabetes mellitus	Yes	4,396 (17.5)	**< 0.0001**
	No	20,702 (82.4)	
	Missing	70 (0.2)	
Hypertension	Yes	9,199 (36.6)	**< 0.0001**
	No	15,841 (63.0)	
	Missing	118 (0.5)	
Angina	Yes	1,055 (4.2)	**0.01**
	No	23,993 (95.5)	
	Missing	110 (0.5)	
Acute myocardial infarction	Yes	1,164 (4.6)	**0.002**
	No	23,901 (95.1)	
	Missing	104 (0.4)	
Stroke	Yes	407 (1.6)	**0.01**
	No	24,681 (98.1)	
	Missing	80 (0.3)	
**Lifestyle and behavioral determinants**			
Smoking (≥100 cigarettes/life)	Yes	13,068 (52.0)	**0.0005**
	No	12,082 (48.0)	
	Missing	18 (0.07)	
Alcohol consumption	Almost daily	4,017(16.0)	**< 0.0001**
	4–5 times weekly	2,546 (10.1)	
	2–3 times weekly	5,139 (20.4)	
	Weekly	2,798 (11.2)	
	2–3 times a month	2,509 (10.0)	
	About once a month	1,655 (6.6)	
	Less than once a month	3,087 (12.3)	
	Never	3,409 (13.5)	
	Missing	10 (0.04)	
Nutritional risk^†^	Low nutritional risk	15,526 (61.7)	**< 0.0001**
	High nutritional risk	8,361 (33.2)	
	Missing	1,281 (5.1)	
Body mass index (kg/m^2^)		28.0 (9.1)	**< 0.0001**
	Missing	99 (0.40)	
Physical activity^†^		131.2 (23.9)	**0.0001**
	Missing	1,038 (4.1)	
Sleep (hours/night)		7.0 (4.3)	0.14
	Missing	54 (0.2)	
**Mental health determinants**			
Depression^†^		5.3 (4.4)	**< 0.0001**
	Missing	101 (0.4)	
Satisfaction with life^†^	Extremely dissatisfied^†^	360 (1.5)	**< 0.0001**
	Dissatisfied	849 (3.4)	
	Slightly dissatisfied	1,727 (6.9)	
	Neutral	493 (2.0)	
	Slightly satisfied	3,726 (15.0)	
	Satisfied	7,761 (31.2)	
	Extremely satisfied	9,984 (31.2)	
	Missing	268 (1.1)	
**Social support determinants**			
Affectional support^†^		86.9 (19.2)	**< 0.0001**
	Missing	75 (0.3)	
Emotional support^†^		80.9 (18.5)	**< 0.0001**
	Missing	228 (1.2)	
Tangible support^†^		81.1 (20.9)	**< 0.0001**
	Missing	52 (0.2)	
Social support^†^		82.7 (18.6)	**< 0.0001**
	Missing	173 (0.7)	
Sense of community belonging	Strongly agree	11,858 (47.1)	**< 0.0001**
	Agree	10,533 (41.9)	
	Disagree	1,273 (5.1)	
	Strongly disagree	216 (0.9)	
	Missing	1,288 (5.1)	
**Living environment determinants**			
Greenness^†^		0.79 (0.17)	**0.04**
	Missing	145 (0.6)	
Material deprivation^†^		−0.018 (0.04)	**−0.01**
	Missing	927 (3.7)	
Social deprivation^†^		0.004 (0.04)	**−0.03**
	Missing	927 (3.7)	
Active living index^†^	1 (=low walkability)	4,424 (17.6)	**0.03**
	2	9,039 (35.9)	
	3	8,254 (32.8)	
	4	2,379 (9.5)	
	5 (=high walkability)	981 (3.9)	
	Missing	91 (0.4)	

* Urban/rural area of residence was defined as follows: Urban core (>100,000 population in large urban area, 10,000–99,000 population in small urban area), Urban fringe (< 10,000 population in small urban area), Urban population (outside of urban core and fringe), Urban secondary core (regions where small and large urban areas were combined).

† The following scales were used in measuring determinants, according to CLSA data collection standards: The AB Screen II Assessment Tool, Physical Activity Scale for the Elderly, Center for Epidemiologic Studies Depression Scale-10, Medical Outcomes Social Support Scale, Normalized Difference Vegetation Index, Active Living Index, Satisfaction with Life Scale, Material and Social Deprivation Index. Bold values indicate statistical significance (*p* ≤ 0.05).

### 2.5 Statistical analysis

Descriptive statistics were calculated for the study outcome and determinants and displayed as means and standard deviations for continuous variables, and frequency and percentages for categorical variables. Bivariate associations for each covariate and the outcome were assessed using ANOVA or Spearman correlation.

#### 2.5.1 Penalized regression method for prioritizing determinants of cognitive function

Research shows that indicators of socioeconomic status, such as income and education, are not interchangeable in relation to health but that each indicator has an independent and unique effect on health (36, 37). To facilitate the use of potentially correlated variables in our study, we utilized a penalization approach to regression which penalizes model parameters to avoid overfitting due to multicollinearity.

Specifically, we used the Elastic Net Regression to select a final model with a unique set of determinants associated with cognitive function in the study sample. The use of Elastic Net Regression in this study was aimed at prioritizing determinants based on the relative magnitude of effect sizes. Traditional regression models with a high number of correlated variables may lead to overfitting the random error which makes it difficult for different parameters to achieve significance (13). Elastic Net Regression is a penalization method for regression that combines Least Absolute Shrinkage And Selection Operator (LASSO) penalty (L1) and ridge penalty (L2). The LASSO (L1) penalty shrinks the parameter coefficients to zero while the ridge (L2) penalty shrinks the correlated parameter coefficients to average (38). Elastic Net groups correlated variables for selection by penalizing the model to prevent arbitrary elimination of correlated variables.

Mixed data consisting of categorical, continuous and binary variables were used for study determinants. Thus, determinants were standardized prior to regression so that the standardized coefficients reflect relative magnitude, regardless of the data type. Continuous variables were not converted to categorical form to avoid loss of power, accuracy and any arbitrary discretization that may impact association with the study outcome. Continuous variables were standardized by subtracting the mean and dividing by the sample standard deviation. Categorical variables were standardized by creating dummy variables which were allowed to enter and leave the model independently. Still, the challenge remains that selection probabilities may differ slightly between categorical and continuous determinants (39). Therefore, the study focused on relative effect sizes rather than directly comparable coefficients and the use of terms such as “double the effect” have been omitted from interpretations.

The Elastic Net Regression was used to select optimal parameters that minimize the average squared errors and achieve the most parsimonious model. All 40 determinants were entered into the model simultaneously. During the regression, determinants were removed from the model until removal no longer improves the model based on the adjusted R-squared. Determinants not retained in the final model were considered not significantly associated with cognitive function and did not improve the model for determining cognitive function in the study sample. The Elastic Net Regression output generated did not produce *p*-values as standard errors are not reliable for penalized estimates. Analyses were performed using SAS/STAT Software Copyright, SAS Institute Inc. SAS and all other SAS Institute Inc. product or service names are registered trademarks or trademarks of SAS Institute Inc., Cary, NC, USA.

## 3 Results

### 3.1 Sample characteristics

Table 2 describes the characteristics of the study sample based on the determinants included in the study. The sample consists of a majority White race, married, educated and living in urban areas. The overall cognition latent construct score was normally distributed with a mean of 99.5 and a standard deviation of 15.2. The interquartile range of the overall cognition latent construct score was 20.4. The lowest overall cognition latent construct score in the sample was 36.6 and the highest score was 169.2. Bivariate associations are shown in the last column of Table 2 where *p* < 0.05 indicated a significant association between the study outcome and listed determinants independently. All determinants were significantly and independently associated with overall cognition, except sex, education, cancer, kidney disease, and sleep.

### 3.2 Penalized regression method for prioritizing determinants of cognitive function

According to the results of the penalized regression, the final model selected 35 of 40 determinants from all seven (7) categories of health determinants as shown in Table 3. Model coefficients from the Elastic Net regression are interpreted in the same way as standardized regression coefficients from ordinary least squares models in both magnitude and direction, with larger effect sizes indicating stronger associations between cognitive function and determinants (Table 3). To summarize, the strongest associations with overall cognition were noted for demographic and socioeconomic, lifestyle and behavioral and mental health determinants. Weaker associations with overall cognition were noted for clinical and living environment determinants. The five determinants removed from the model include: total cholesterol, cancer, kidney, angina and sleep.

**Table 3:** Model estimates produced by the Elastic Net Regression for the association between cognitive function and determinants of health, Canadian Longitudinal Study of Aging Baseline Cohort 2015 (CLSA).

**Determinants of health**		**Standardized beta coefficient**
**Demographic and socioeconomic determinants**		
Age (years)	45–54	1.64
	55–64	−0.56
	65–74	−0.27
	75 and older	*NS^†^*
Race	White (vs. Non-White)	2.32
Marital status	Single	0.40
	Married/common-law	1.89
	Widowed	*NS^†^*
	Divorced	*NS^†^*
	Separated	*NS^†^*
Sex	Female (vs. Male)	−0.53
Urban/rural area of residence^‡^	Rural	−0.50
	Urban core	0.70
	Urban fringe	*NS^†^*
	Urban population	3.06
	Urban secondary core	*NS^†^*
Education	Less than secondary	−1.64
	Some post secondary	0.04
	Graduated secondary	1.45
	Post secondary	*NS^†^*
Annual household Income	< $20,000	0.35
	$20,000–49,999	2.02
	$50,000–99,999	0.04
	$100,000–149,999	−1.29
	$150,000 or more	−1.70
Immigrant status	Yes (vs. No)	−2.59
Access to primary care	Has a primary physician	0.80
**Clinical determinants**		
Blood systolic pressure (mmHg)		−0.02
Blood diastolic pressure (mmHg)		0.04
Non-HDL (mmol/L)		0.06
Total cholesterol (mmol/L)^†^		*NS* ^†^
HBA1c (%)		−0.76
**Chronic disease determinants**		
Heart disease	Yes	−1.18
Peripheral artery disease	Yes	−2.02
Cancer	Yes	*NS^†^*
Kidney disease	Yes	*NS^†^*
Diabetes mellitus	Yes	−0.17
Hypertension	Yes	−0.30
Angina	Yes	*NS^†^*
Stroke	Yes	−1.69
Acute myocardial infarction	Yes	−0.22
**Lifestyle and behavioral determinants**		
Smoking (≥100 cigarettes/life)	Yes (vs. No)	−0.19
Alcohol consumption	Almost daily	−0.91
	4–5 times weekly	−0.90
	2–3 times weekly	0.78
	Weekly	−1.06
	2–3 times a month	*NS^†^*
	About once a month	*0.52*
	Less than once a month	*NS^†^*
	Never	1.44
Nutritional risk^‡‡^	High nutritional risk (vs. Low)	−2.09
Body mass index		0.02
Physical activity^‡‡^		0.003
Average hours of sleep/night		*NS^†^*
**Mental health determinants**		
Depression^‡‡^		−0.17
Satisfaction with life^‡‡^	Extremely dissatisfied	−4.16
	Dissatisfied	−0.65
	Slightly dissatisfied	0.54
	Neutral	−1.02
	Slightly satisfied	0.26
	Satisfied	1.02
	Extremely satisfied	−0.17
**Social support determinants**		
Affectional support^‡‡^		−0.02
Emotional support^‡‡^		0.01
Tangible support^‡‡^		0.03
Social support^‡‡^		0.01
Sense of community belonging	Strongly agree	*NS*
	Agree	0.45
	Disagree	−2.57
	Strongly disagree	−2.07
**Living environment**		
Greenness^‡‡^		0.001
Material deprivation^‡‡^		−0.001
Social deprivation^‡‡^		−0.001
Active Living Index^‡‡^	Low active living environment	−0.41

^*^ Categorical variables were centered and thus entered into models as dummy variables, where 1 indicated the category of interest and 0 indicated all other categories in the variable.

† Cells labeled “NS” indicate categories or variables that were Not Significant and were thus not selected in the model.

‡ Urban/rural area of residence was defined as follows: Urban core (>100,000 population in large urban area, 10,000–99,000 population in small urban area), Urban fringe (< 10,000 population in small urban area), Urban population (outside of urban core and fringe), Urban secondary core (regions where small and large urban areas were combined).

‡‡ The following scales were used in measuring determinants, according to CLSA data collection standards: The AB Screen II Assessment Tool, Physical Activity Scale for the Elderly, Center for Epidemiologic Studies Depression Scale-10, Medical Outcomes Social Support Scale, Normalized Difference Vegetation Index, Active Living Index, Satisfaction with Life Scale, Material and Social Deprivation Index.

Demographics and Socioeconomic determinants demonstrating the greatest associations with cognition were race, immigration, urban/rural area of residence and income. The Clinical Determinant demonstrating the greatest negative association with cognition was HBA1c. Three Chronic Disease Determinants were retained in models, with peripheral artery disease and stroke demonstrating the greatest negative association with cognition. Five Lifestyle and Behavioral Determinants were retained in models, with poor nutritional risk demonstrating the greatest negative association with cognition. Mental Health Determinants were retained, with extreme dissatisfaction with life demonstrating the greatest negative association with cognition. Five Social Support Determinants were retained in models with a poor sense of community belonging demonstrating the greatest negative association with cognition.

**Figure 1:**
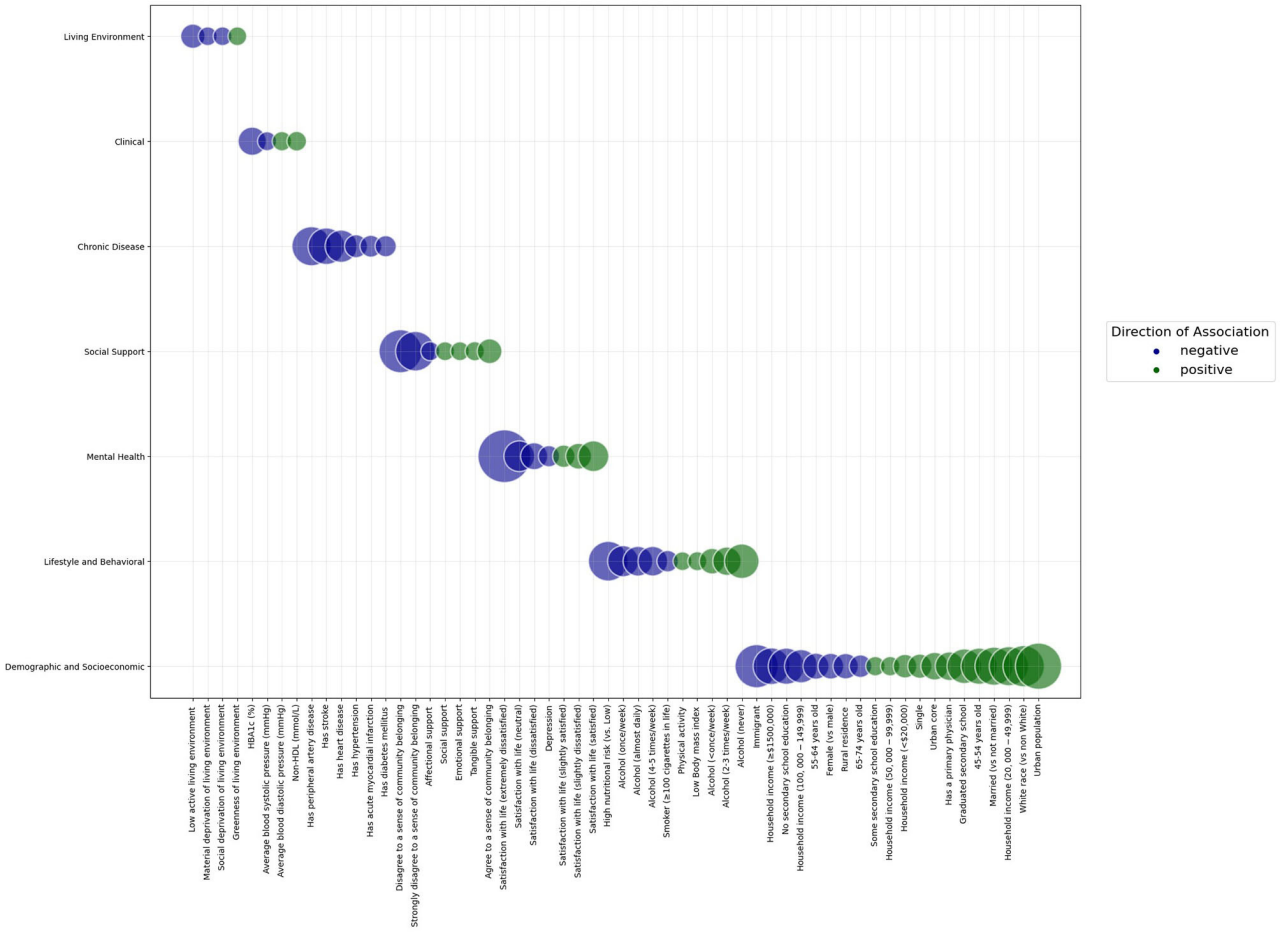
Bubble graph demonstrating the standardized effect sizes of the association between study determinants and cognitive function in a sample of adults aged 45 years and older from the Canadian Longitudinal Study of Aging Baseline Cohort. Categories of determinants are shown on the y axis. Study determinants are shown on the x axis. The size of the bubbles represents the magnitude of association between the determinant and cognitive function. The color of the bubbles represents the direction of association between the determinant and cognitive function; blue bubbles represent a negative association, while green bubbles represent a positive association. Within each category of determinants, bubbles are arranged from negative to positive associations.

Figure 1 summarizes the effect sizes for the association between study determinants and cognitive function in the final model.

## 4 Discussion

Using a machine learning regression approach in a sample of healthy adults, our study found that the determinants demonstrating the strongest associations with healthy cognitive function, were demographic and socioeconomic factors, and lifestyle and health behaviors. Overall, better cognitive function was noted for adults who were White race, younger, married, male, living in urban areas and had a higher education level. Conversely, worse cognitive function was noted for those who had chronic disease, depression, elevated systolic blood pressure and HBA1c, smoked cigarettes, lower physical activity and sleep. Results of this study do not discount the importance of clinical care or the living environment in the prioritization process for addressing cognitive health. Rather, study findings suggest that in adults who have not yet experienced cognitive impairment, demographic and socioeconomic factors along with behavioral and lifestyle factors play a substantive role in determining healthy cognitive function.

The supervised machine learning method used in this study aimed to reduce a large set of determinants into a smaller set representing only key features of the data. Notably, the model retained almost 90% of input determinants. Indeed, research has indicated the complexity of factors that contribute to health outcomes with the County Health Rankings model adding seven new determinants to its original model in 2014 (8, 40). Results of this study confirm that overall cognition is impacted by multiple groups of determinants acting simultaneously, therefore, unimodal interventions may not be the most effective method for addressing cognitive health in middle aged to older adults. This conclusion emphasizes the need to consider urgent population level action such as the “Health in All Policies” (HiAP) approach in Canada, where health is considered by all policy makers, including those not directly involved in healthcare such as education, housing and food security (41).

Of note, the relative magnitude of effect sizes was greatest for demographic and socioeconomic factors and lifestyle and behavioral factors. Literature has confirmed the strong influence of education, diet, and social isolation independently on cognitive function (42–44). However, few studies have included the wide range of determinants addressed in the current study. Findings of this study confirm the strong collective influence of socioeconomic and lifestyle determinants on cognitive function. Adding to the existing literature, results of this study reveal the high contribution of race, immigration, satisfaction with life and community belonginess to cognitive function in middle-aged and older adults. Future studies addressing dementia prevention should consider such factors as strong determinants rather than nuisance confounders in cognitive function associations.

According to the 2017 Lancet Commission Report, about 40% of worldwide dementia could be prevented or delayed by addressing nine modifiable risk factors: less education, hypertension, hearing impairment, smoking, obesity, depression, physical inactivity, diabetes, and low social contact (45). In the updated 2020 Report, three more risk factors were included: excessive alcohol consumption, traumatic brain injury, and air pollution (46, 47). As data analytics expand our computational abilities, new risk factors for dementia are emerging rapidly and the list of modifiable risk factors is expected to grow. Considering the limited resources allocated toward prevention policy, our study takes an important step by identifying those key determinants, which when adequately addressed through effective interventions, are more likely to contribute significantly to improving cognitive health at the population level.

The Finnish Geriatric Intervention Study to Prevent Cognitive Impairment and Disability (FINGER) trial, completed more than a decade ago, was one of the first multidomain lifestyle interventions to establish a beneficial effect on cognitive outcomes in at-risk elderly individuals regardless of demographic and socioeconomic characteristics (48). It is important to note that the success of this trial may be attributed to the at-risk, elderly population targeted for the intervention. Subsequent studies that do not include at-risk populations have failed to replicate the success of the FINGER trial (49–51). Taken together, the application of these findings have been summarized in the widely recognized publication ‘Sick individuals and sick populations' by Geoffrey Rose (52). Indeed, addressing modifiable risk factors in at-risk individuals will reveal beneficial effects for reducing risk of dementia in a small proportion of individuals. However, an early population-based approach of addressing socioeconomic and lifestyle factors in the healthy population, may have a greater impact on improving cognitive health and preventing the onset of dementia in the entire population.

In accord with our research findings, key studies have confirmed that socioeconomic factors were similar in importance for reducing premature mortality compared to twenty five other major modifiable risk factors (53). Additionally, studies on both high-income and low- and middle-income countries have demonstrated a greater distribution of disease in groups with lower socioeconomic status (54, 55). Nevertheless, socioeconomic status is constantly referred to as a non modifiable risk factor in disease prevention strategies (53). Findings from our study emphasize that socioeconomic status should be a key component of future interventions for maintaining cognitive health. Although socioeconomic factors such as income may not be immediately altered, health authorities can directly address this issue through targeted policies and interventions aimed at reducing income related inequalities and the impact on cognitive health.

An important caveat in the findings was the lack of association between education and cognitive function in bivariate analysis, followed by the negative association between income and cognitive function in the model analysis. Suggested reasons for the results are speculative but may be attributed to the shared causal pathway between education and income (56). The current study shows a positive association between higher education and cognitive function as seen in other studies (57). Although this should not negate the impact of income, there may be some interactive effects between measures of socioeconomic status not tested in the current model that are not purely correlative. Another reason could be the high levels cognitive function of the sample, suggesting a possible ceiling effect of income after controlling for other socioeconomic factors (58). Although the disentanglement of socioeconomic status indicators was beyond the scope of this study, future studies should consider the findings observed here and investigate in future studies.

The issue of multicollinearity in traditional regression produces biased standard errors and can cause some significant variables to appear nonsignificant (59, 60). To determine and address the problem of multicollinearity, advanced regression methods such as the one used in this paper are required. Our study is an important step toward the use of advanced methodologies for examining the influence of multiple correlated determinants of health simultaneously. Consequently, findings from this study can be used to develop novel interventions for preventing cognitive decline. Systematic reviews have confirmed that multidomain interventions are associated with improvements in cognitive function in the elderly (61, 62). Indeed, results support multidimensional interventions but additionally suggests targeting these interventions toward subpopulations most in need of preventive care such as those with poor mental health, low social support or low socioeconomic status.

### 4.1 Study limitations

The main limitation to this study is the lack of external generalizability beyond the study sample due to the lack of an external validation dataset. While the sample size may be sufficient, our sample consists of healthy adults who were majority White and educated with well-preserved cognitive function. The social and environmental contexts for this subgroup may differ significantly from the general population. Furthermore, the use of elastic net models does not remove all associated study biases. For example, age 75 years and older was removed from the final model. This finding may be the result of survival or attrition bias, that is, if a selective group of ‘dementia-free, noninstitutionalized adults' represent the group aged 75 years and older, the relationship between cognitive function and age may be underestimated or in this case removed from the model entirely. Additionally, the use of cross-sectional data prohibits the temporal sequence necessary to establish causality between determinants and cognitive function. It is important to note that this is the most parsimonious model selected using the study outcome and determinants in this study sample. This does not mean that it is the only possible model; other possible models may be selected using alternate methods. Finally, effect sizes are to be interpreted with caution due to standardization and shrinkage of parameters in the model. This study did not focus on quantification of effect sizes for determinants but examined their correlated order for prioritization purposes.

## 5 Conclusion

In summary, the current study demonstrates that healthy cognitive function in older adults is not solely influenced by one group of determinants, but by multiple groups of determinants acting simultaneously. Further, demographic and socioeconomic, as well as lifestyle and behavioral, determinants should be prioritized in targeted interventions toward improving cognitive health; for example, addressing nutritional knowledge in low-income communities or community engagement in immigrant subgroups. The methodology used in this paper can be applied to a wide range of existing healthcare data allowing, in the future, for more in depth exploration of determinants of health and evaluating model performance.

## Data availability statement

This research was made possible using the data/biospecimens collected by the Canadian Longitudinal Study on Aging (CLSA). Funding for the Canadian Longitudinal Study on Aging (CLSA) is provided by the Government of Canada through the Canadian Institutes of Health Research (CIHR) under grant reference: LSA 94473 and the Canada Foundation for Innovation, as well as the following provinces, Newfoundland, Nova Scotia, Quebec, Ontario, Manitoba, Alberta, and British Columbia. This research has been conducted using the CLSA dataset (Baseline Comprehensive version 7.0), under Application Number (2109031). The CLSA is led by Drs. Parminder Raina, Christina Wolfson, and Susan Kirkland. Data are available from the Canadian Longitudinal Study on Aging (www.clsa-elcv.ca) for researchers who meet the criteria for access to de-identified CLSA data.

## Ethics statement

The requirement of ethical approval was waived by Western University Research Ethics Board for the studies involving humans. The studies were conducted in accordance with the local legislation and institutional requirements. The participants provided their written informed consent to participate in this study.

## Author contributions

SS: Conceptualization, Data curation, Formal analysis, Investigation, Methodology, Software, Visualization, Writing—original draft, Writing—review & editing. SZ: Conceptualization, Data curation, Investigation, Writing—review & editing. KR: Conceptualization, Funding acquisition, Investigation, Project administration, Supervision, Writing—review & editing. VH: Conceptualization, Funding acquisition, Investigation, Supervision, Writing—review & editing. SF: Conceptualization, Investigation, Project administration, Supervision, Writing—review & editing.
