# Corrigendum: Prioritizing determinants of cognitive function in healthy middle-aged and older adults: insights from a machine learning regression approach in the Canadian longitudinal study on aging

**DOI:** 10.3389/fpubh.2024.1372914

**Published:** 2024-02-12

**Authors:** Sarah Singh, Shiran Zhong, Kem Rogers, Vladimir Hachinski, Stephanie Frisbee

**Affiliations:** ^1^Robarts Research Institute, University of Western Ontario, London, ON, Canada; ^2^Department of Geography, University of Western Ontario, London, ON, Canada; ^3^Department of Anatomy and Cell Biology, Schulich School of Medicine and Dentistry, University of Western Ontario, London, ON, Canada; ^4^Department of Clinical Neurological Sciences, and Epidemiology and Biostatistics, Schulich School of Medicine and Dentistry, University of Western Ontario, London, ON, Canada; ^5^Department of Pathology and Laboratory Medicine, and Epidemiology and Biostatistics, Schulich School of Medicine and Dentistry, University of Western Ontario, London, ON, Canada

**Keywords:** cognitive function, determinants of health, dementia prevention, machine learning, CLSA

In the published article, there was an error in the Data Availability statement. The incorrect statement was used (“The original contributions presented in the study are included in the article/supplementary material, further inquiries can be directed to the corresponding author”). The data source for this study (CLSA) requires the inclusion of their acknowledgment statement in this section. The correct Data Availability statement appears below.

## Data availability statement

“This research was made possible using the data/biospecimens collected by the Canadian Longitudinal Study on Aging (CLSA). Funding for the Canadian Longitudinal Study on Aging (CLSA) is provided by the Government of Canada through the Canadian Institutes of Health Research (CIHR) under grant reference: LSA 94473 and the Canada Foundation for Innovation, as well as the following provinces, Newfoundland, Nova Scotia, Quebec, Ontario, Manitoba, Alberta, and British Columbia. This research has been conducted using the CLSA dataset (Baseline Comprehensive version 7.0), under Application Number (2109031). The CLSA is led by Drs. Parminder Raina, Christina Wolfson, and Susan Kirkland. Data are available from the Canadian Longitudinal Study on Aging (www.clsa-elcv.ca) for researchers who meet the criteria for access to de-identified CLSA data.”

The authors apologize for this error and state that this does not change the scientific conclusions of the article in any way. The original article has been updated.

